# COVID-19 Response in Latin America

**DOI:** 10.4269/ajtmh.20-0765

**Published:** 2020-09-15

**Authors:** Patricia J. Garcia, Alex Alarcón, Angela Bayer, Paulo Buss, German Guerra, Helena Ribeiro, Karol Rojas, Rocío Saenz, Nelly Salgado de Snyder, Giorgio Solimano, Rubén Torres, Sebastián Tobar, Rafael Tuesca, Gilma Vargas, Rifat Atun

**Affiliations:** 1 School of Public Health, Cayetano Heredia University, Lima, Peru;; 2 Alianza Latinoamericana de Salud Global (ALASAG), Latin American Alliance for Global Health;; 3 School of Public Health “Dr. Salvador Allende G.”, Faculty of Medicine, University of Chile, Santiago, Chile;; 4 The Oswaldo Cruz Foundation, Rio de Janeiro, Brazil;; 5 Global Health Program, National Institute of Public Health, Cuernavaca, Mexico;; 6 School of Public Health, University of São Paulo, São Paulo, Brazil;; 7 School of Public Health, Costa Rica University, San Pedro, Costa Rica;; 8 SALUD University, Buenos Aires, Argentina;; 9 Fiocruz Center of Global Health, Rio de Janeiro, Brazil;; 10 Department of Public Health, Universidad del Norte, Barranquilla, Colombia;; 11 National School of Public Health, University of Antioquia, Medellín, Colombia;; 12 Harvard T.H. Chan School of Public Health, Harvard University, Boston, Massachusetts

## Abstract

Effective management of a pandemic due to a respiratory virus requires public health capacity for a coordinated response for mandatory restrictions, large-scale testing to identify infected individuals, capacity to isolate infected cases and track and test contacts, and health services for those infected who require hospitalization. Because of contextual and socioeconomic factors, it has been hard for Latin America to confront this epidemic. In this article, we discuss the context and the initial responses of eight selected Latin American countries, including similarities and differences in public health, economic, and fiscal measures, and provide reflections on what worked and what did not work and what to expect moving forward.

## INTRODUCTION

On March 11, 2020, the WHO declared SARS-CoV-2 a pandemic.^1^ The first case of SARS-CoV-2 infection in Latin America (LA) was reported on February 26 in Brazil, and the first death from COVID-19 in LA was reported on March 7 in Argentina. By March 19, every country in LA had reported SARS-CoV-2 infections.^2^

The epidemic initially affected higher income population groups returning from abroad and their close contacts but spread rapidly to lower income populations that have less access to health services. Effective management of a pandemic due to a respiratory virus requires public health capacity for a coordinated response for mandatory restrictions (such as school closures, lockdowns, quarantines, or curfews), voluntary restrictions (e.g., social distancing and use of masks), large-scale testing to identify infected individuals, isolation of infected cases, and tracing and quarantine of the contacts of infected cases. In addition, a public health response must include rigorous surveillance and real-time monitoring of the trajectory of the epidemic and efficient management of human resources for health care and related essential services and of healthcare supplies (e.g., personal protective equipment (PPE), medicines, and ventilators) to create surge capacity and manage those at risk.^3^ However, mandatory and voluntary restrictive measures, such as confinement and social distancing, are particularly challenging in LA because of cultural norms, characterized by close personal relations and extended families.^4^

## THE CONTEXT

COVID-19 poses a major risk to LA. Although countries in the region share many economic, political, social, cultural and health system similarities, they are also diverse, given varying levels of universal health coverage and different multi-sectoral responses to address social determinants of health.^5,6^ Resilience of health systems to economic, political, and epidemiological shocks also vary, with many LA nations experiencing long-lasting deteriorations in several population health outcomes following economic and political shocks.^7–9^

However, what is common to all the LA is wide inequalities in income, effective access to healthcare services, and health outcomes. Latin America is the most inequitable region in the world, as measured by the Gini Index.^10^ On average, 53% of LA’s working population is engaged in informal work with precarious income and social protection. In some countries like Peru, the level of informality can be as high as 70%.^11^ In the region, around 185 million people’s incomes are below the poverty threshold, of whom 66 million live in extreme poverty.^12^

Congregate settings favorable for increased transmission of the virus are present in LA because of rapid urbanization, which has produced crowded megacities such as Bogota in Colombia, Buenos Aires in Argentina, Lima in Peru, Mexico City in Mexico, and Rio de Janeiro and Sao Paulo in Brazil, and widespread poverty.^12^ This situation has worsened since 2015 following the migration of millions of Venezuelans to different LA countries. The emergence of migrant communities with inadequate living conditions, jobs, and access to health services has further stretched the capacity of the region’s health systems, which suffer from low levels of investment in public health and high levels of corruption.^13–15^

Political instability, also common in LA countries, has hampered the leadership needed to guide responsible and timely actions to confront major events, as has the structure of segregated health systems and the low investment in these systems. Health systems in LA are typically composed of a small private sector for those with private insurance, a relatively well funded social security system for salaried workers and their families, and a publicly funded and operated ministry of health system serving lower income population groups with inadequate access to effective health services.^16^ Decentralization of health-related funding and decision-making to states, provinces, municipalities, or social insurance organizations has further fragmented health systems and created challenges for mounting an integrated and coordinated health response. Relatively low levels of investment in already stretched public health systems and lack of sufficient trained human health resources, mainly concentrated in capital cities,^17^ coupled with rapid population aging and rising prevalence of chronic diseases,^18^ have led to weak public primary health care and hospital systems with suboptimal capacity of intensive care units. This represents a difficult context for confronting the pandemic (Table 1).

**Table 1:** 2017 Gross domestic product; healthcare expenditure per capita; number of ICU beds, ventilators, and physicians (per 100,000 inhabitants and total) in eight selected Latin American countries, compared with the United States

Country	GDP* (nominal) per capita	Current health expenditure as % of GDP†	Number of ICU beds/100,000 inhabitants (total)	Number of ventilators/100,000 inhabitants (total)	Number of ICU specialists/100,000 inhabitants (total)
Argentina‡	$14,508	9.12	19.2 (8,444)	20.4 (9,000)	3.0 (1,320)
Brazil§	$9,881	9.47	26 (55,000)	31 (65,411)	2.4 (5,112)
Chile^‖^	$15,001	8.98	12.1 (2,300)	8 (1,520)	0.5 (95)
Colombia¶	$6,429	7.23	10.7 (5,349)	11 (5,500)	2.4 (1,200)
Costa Rica#	$11,573	7.33	2.74 (140)	8.8 (450)	0.78 (41)
Ecuador**	$6,214	8.26	2.7 (1,183)	3.8 (663)	2.85 (436)
Mexico††	$9,224	5.52	3.37 (4,291)	4.86 (6,175)	1.56 (1,929)
Peru‡‡	$6,723	5.00	2.64 (820)	2.90 (900)	2.25 (700)
United States§§	$59,939	17.06	34.7 (114,000)	60.8 (200,000)	10.34 (34,000)

GDP = 2017 gross domestic product; ICU = intensive care unit.

* GDP = 2017 Gross domestic product https://www.worldometers.info/gdp/gdp-per-capita/ (accessed August 23, 2020).

† https://apps.who.int/nha/database/Select/Indicators/en (accessed August 23, 2020).

‡ Reporte Diario desde el Ministerio de Salud (April 20, 2020) Dr. Arnaldo Medina, Subsecretario de Calidad en Salud www.infobae.com (accessed August 23, 2020); Entrevista al Ministro de Salud Dr. Ginés Gonzalez García (April 19, 2020) en programa televisivo “Antes de Mañana,” entrevistador Antonio Laje. www.a24.com (accessed August 23, 2020).

§ Scheffer, M. (coord). Demografia Médica no Brasil 2018. Departamento de Medicina Preventiva. Faculdade de Medicina da Universidade de São Paulo; Conselho Regional de Medicina de São Paulo; Conselho Federal de Medicina, São Paulo. 2018. 286 pág. ISBN 978-85-87077-55-4; ICU and ventilators. Boletim Epidemiológico do Ministério da Saúde. N.7. https://www.saude.gov.br (accessed August 23, 2020); https://covid-insumos.saude.gov.br/paineis/insumos/painel.php (accessed August 23, 2020); Personal communication with COE (Centro de Operações de Emergência) -COVID-19. Ministério da Saúde. Brasil.

‖ https://www.emol.com/noticias/Nacional/2020/03/22/980632/Camas-criticas-Chile-caracteristicas.html (accessed August 23, 2020).

¶ https://www.eltiempo.com/datos/total-de-camas-de-cuidado-intensivo-en-colombia-478076; https://www.sciencedirect.com/science/article/pii/S0122726217300137 (accessed August 23, 2020); Periódico “el tiempo” Respiradores, una carrera vital para enfrentar el coronavirus. 20 de Abril 2020 [Internet] https://www.eltiempo.com/salud/respiradores-hay-suficientes-en-colombia-485904 (accessed August 23, 2020).

# https://delfino.cr/2020/04/ccss-advierte-80-de-las-camas-para-cuidados-intensivos-estan-ocupadas (accessed August 23, 2020); Personal communication with Dr. Mario Ruiz Cubillo, Medical Manager of the CostaRican Social Security Fund. CCSS.

** https://saludconlupa.com/series/coronavirus/latinoamerica-en-cuidados-intensivos/ (accessed August 23, 2020) https://www.ecuadorencifras.gob.ec/documentos/web-inec/Estadisticas_Sociales/Recursos_Actividades_de_Salud/Publicaciones/Anuario_Rec_Act_Salud_2011.pdf (accessed August 23, 2020).

†† https://www.informador.mx/mexico/Mexico-enfrenta-pandemia-con-deficit-de-camas-hospitalarias-20200326-0141.html (accessed August 23, 2020); https://www.gob.mx/cms/uploads/attachment/file/529236/CPM_Pulso_de_la_Salud__28ene20.pdf (accessed August 23, 2020) https://www.anmm.org.mx/GMM/2018/n3/GMM_154_3_342-351.pdf (accessed August 23, 2020).

‡‡ Peru: https://saludconlupa.com/entrevistas/sin-respiro-unidades-de-cuidados-intensivos-necesitan-700-medicos/ (accessed August 23, 2020).

§§ https://sccm.org/Blog/March-2020/United-States-Resource-Availability-for-COVID-19 (accessed August 23, 2020).

## INITIAL RESPONSE TO COVID-19 PANDEMIC IN EIGHT SELECTED LATIN AMERICAN COUNTRIES

**Figure 1.**
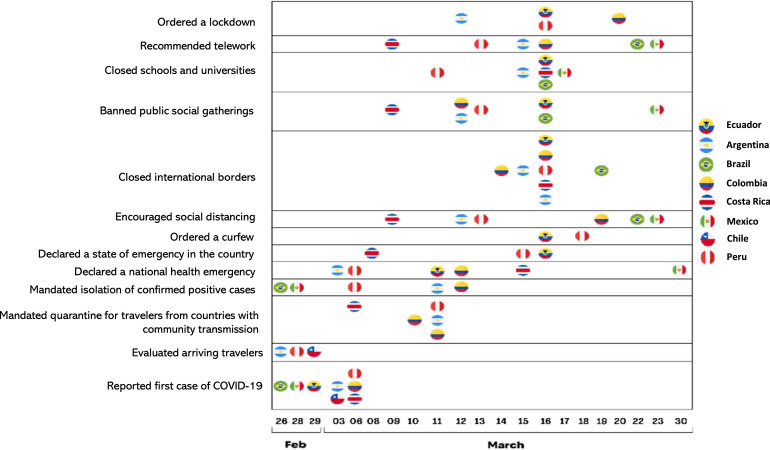
Timeline for the initial major responses to the COVID-19 pandemic in eight Latin American countries, by country.

The evolution of the pandemic and the nature and stringency of the response varied by country. Most countries in LA responded with multiple, early well-sequenced measures (see Figure 1, Table in Supplemental Materials).

### Argentina.

Argentina confronted the pandemic with a relatively recent government, which started in December 2019. Although this country has one of the most robust healthcare systems in the region,^19^ it was clear that its systems might not be sufficient for facing the pandemic; thus, the central government decided to act early.

On February 26, the country initiated preventive assessment and self-reporting measures for travelers coming from Italy and other affected countries. On March 3, the first COVID-19 case in Argentina was reported. This prompted the introduction of voluntary isolation of health workers with COVID-19 symptoms and public and private sector workers returning from countries with community transmission and quarantine of contacts of both groups. Days later, all public events were banned. On March 12, the Presidential Necessity and Urgency Decree expanded the health emergency response and enabled the adoption of new measures to contain the spread of SARS-CoV-2. Several toll-free lines were established nationally and in provinces for citizens to inquire about COVID-19 and the government recommendations. Schools, universities, and national parks were closed shortly after. Remote work was introduced for all public sector workers, with the exception of those providing essential services, and recommended for private companies. On March 26, the Argentine government imposed a compulsory quarantine period and near total lockdown of the economy and public spaces.

During the implementation of COVID-19 prevention and control measures, Argentina has had a broad-reaching consultative process in place. This process has included dialogue at the national level and with provincial governors and municipal mayors, across political parties, with the participation of public health experts, trade unions, actors from the private and public sectors, and considerable community engagement. Close coordination of lockdown measures between the central government, governors, and mayors in Argentina has been key in the response. There has also been an important effort to reach the public with the right information, working with journalists and national and provincial authorities to increase their understanding of COVID-19, and to have a consensus on the messages provided to the population. Probably, as a result of the efforts described, Argentina is reported to be among the countries in the region whose population had the best adherence to the national lockdown.^20^

### Brazil.

Although Brazil has a large health system that provides universal access with strong primary health care for all Brazilian citizens, the pandemic found the country with a new president with a popularity which had been declining since his election. In addition, major investigations into corruption scandals have further eroded citizens’ trust in politicians in Brazil.

In preparation for the pandemic on January 20, the Ministry of Health created a Public Health Emergency Operations Center, and on February 3, the country declared COVID-19 a Public Health Emergency of National Importance. On February 26, the first COVID-19 case in Brazil was confirmed, and by March 22, COVID-19 had spread in all 26 states, Brasilia, and Rio de Janeiro.

Outside of the initial actions just described, there were few public health or disease control measure decisions made at the central level. Instead, these actions took place at the municipal and state levels. São Paulo took actions first, closing schools on March 16 and issuing guidance to “stay at home” to work remotely and closing all museums, public buildings, parks, and beaches on March 20. Pharmacies and grocery stores were allowed to stay open, and restaurants were only allowed to deliver food. Other cities and states followed suit with similar measures shortly after.

Federal and state governments introduced measures to reduce the economic and social impacts of the crisis by expanding the number of families entitled to social transfers and other social benefits. Despite these immediate measures, millions in Brazil, especially low-income citizens in urban areas who live hand to mouth and workers and students who cannot work remotely and need resources for food, rent, utilities, medicines, and debts, are suffering catastrophic declines in well-being and quality of life and getting infected with COVID-19.

Additionally and importantly, there were political tensions and conflicting messages to the public. Although state governors, mayors, and Ministry of Health officials were urging people to stay home and maintain social distancing, the president was dismissing the danger posed by the virus and encouraging people to continue working to keep the economy from collapsing. Given these contradictory views, the Supreme Court declared that decisions about quarantine and other COVID-19–related restrictions should be made by state governors and mayors.^21,22^ With no central coordination, the disease has become widespread in Brazil and made control at the national level very difficult.

### Chile.

The pandemic arrived in Chile at a moment of political polarization. Since October 2019, Chile has experienced major social and political unrest with large protests in opposition to the economic and social model, in particular inequities in health, education, and other socioeconomic conditions, which has led to underserved communities. Chile has a mix of public and private insurance with substantial inequalities between high-income participants in the private system and the large majority covered by social insurance and tax-funded public health services.^23^

On March 3, the first COVID-19 case in Chile was reported. COVID-19 cases started in the Santiago capital metropolitan area and spread out of the wealthy areas of Santiago to low-income neighborhoods where many residents could not afford to work from home. Infections quickly began to increase in these low-income neighborhoods. Later on, outbreaks started in other regions in the country.

Early in March, a group of experts convened by the government identified challenges to be addressed: delays in reporting of molecular test results; inadequate isolation of infected people and contact tracing; inadequate quarantine of travelers coming from other countries, such as the United States; low levels of medical supplies and personal protective equipment; and lack of information about new cases and mortality. As a result of the experts’ recommendations, national border closure measures were implemented and laboratory screening capacity was expanded by including all of the country’s institutions with PCR equipment and supplying reagents to the laboratory network of universities/institutes. As a result, Chile has one of LA’s highest rates of COVID-19 testing.^24^

On March 22, a nightly curfew was implemented throughout Chile, but no national lockdown was established. Instead, quarantines were established locally in different cities and neighborhoods. Protests sparked in late May, mainly in Santiago, because of food shortages in certain sectors of the population. Soon after, the city of Santiago started a mandatory quarantine because of increased COVID-19 cases, and similar situations were extended to most of the largest cities in Chile.

Because of pressure from the Chilean Medical Association, scientific societies, universities, the Chilean Association of Mayors, and civil society organizations, the central government has gradually adopted different measures to improve surveillance and better manage the pandemic according to international recommendations. The government also released economic measures that sought to benefit the most vulnerable families in the country, small businesses, and employment.^25^ Communication campaigns have also been developed and have evolved through the pandemic from “let’s take care of each other” to “you can be the next one,” focusing on population members’ responsibility to comply with sanitary measures.

### Colombia.

Colombia has experienced years of unrest with precarious peace between opposing factions. Millions of people have been forcibly displaced from their homes and communities because of violence, and the majority of them have resettled in extremely precarious conditions, placing them at higher risk of infection and bad outcomes. The pandemic found the country with a fragile state of peace and a fragmented health system which, despite reforms, has not been able to achieve universal health care and access.^5^

On March 6, the first COVID-19 case in Colombia was diagnosed in a person who traveled from Italy. Because of the increasing number of cases, on March 12, the Ministry of Health and Social Protection declared “health emergency in the country because of the COVID-19” and introduced isolation and quarantine measures, banned events with more than 500 people, requested that state governors and mayors assess transmission risks, and ordered commercial establishments and stores to implement hygiene and sanitation measures for users and workers. On March 13, international tourist ships were banned from docking in Colombia. Four days later, the president declared a state of emergency and added measures such as the closure of schools, restaurants, and bars and the order that people older than 70 years should stay at home unless buying essential food or health products.

After several major protests in Colombian prisons due to large coronavirus outbreaks, the government in an attempt to alleviate the burden on prisons, released a decree offering conditional release or transfer to house arrest for inmates under specific conditions.^26^ The power of municipal and regional authorities was removed to assure the measures were taken nationally, which resulted in some civil protests. As in other LA countries, the pandemic has particularly impacted poorer communities. The Colombian government has implemented a number of policies toward social protection and economic measures in an attempt to improve the situation.^25^

Since the start of the pandemic, the president, together with the technical team, has presented updates about the pandemic in the country almost daily; however, there has been no specific plan or activity for risk communication by target audience, competencies, and scope.

### Costa Rica.

Costa Rica is a country that has achieved political stability and sustained economic growth over the past 25 years, although financial challenges and social inequalities still remain. It has one of the best health systems in the region, provides universal health care to all citizens, and has a well-established primary care infrastructure. Costa Rica is characterized as a society that follows instructions and acts with responsibility.

On March 6, the first case of COVID-19 was reported. A few days later, following an increase to nine confirmed COVID-19 cases, the health authorities and the National Emergency Commission declared a national yellow alert status. This alert enabled the systematic and interinstitutional mobilization of resources and the activation of emergency operations centers. On March 9, remote work was introduced in the public sector and recommended for the private sector; large meetings were canceled; and distancing measures were introduced for leisure and sporting events. On March 12, schools without an adequate drinking water supply and where an educator tested positive were closed and international travel by public sector employees was banned. Subsequent measures included restrictions on the entry of foreigners and reductions in the capacity of shopping centers, cinemas, and theaters by 50%. On March 23, the government introduced a nightly curfew from 10 pm to 5 am for private cars and ordered the closure of beaches, temples, and religious services.

Costa Rica used its social sector institutions to mount a multi-sectoral response to the COVID-19 crisis. Economic measures, employment protections, and temporary tax and insurance relief were introduced to protect workers on March 14. Other efforts were taken to ensure the supply of clean water and reduce risks for the most vulnerable populations such as minors, older adults, indigenous population, homeless people, and families in poverty.

The communication strategy has been led primarily by the Minister of Health, who has focused his messages on technical issues, informational updates, and the promotion of protection measures and behavior changes to avoid risk of infection and transmission.

### Ecuador.

Ecuador is facing the COVID-19 pandemic amid a major political crisis, given a government with low popularity, weak political opposition, allegations of corruption, and social discontent. Ecuador enjoyed strong economic growth and poverty reduction in the last decade, thanks to high oil prices. However, a decrease in oil prices in 2019 magnified persistent structural problems including widespread inequalities, adversely affected the Ecuadorian economy, and exacerbated the pandemic situation. The government sought approximately $500 million from the International Monetary Fund at the end of 2019, to support the economy. Regarding the health system, the country has undertaken important reforms in search of universality and equity; however, it continues fragmented and does not guarantee universal access.

On February 29, the first case of COVID-19 in Ecuador was reported in a citizen who traveled from Spain to Guayaquil. The first cluster of cases emerged in early March. By March 23, there were confirmed cases in at least three provinces and close to that date, the first death in Guayaquil. From Guayaquil, the epicenter moved to Quito, the capital.

On March 11, the government declared a “State of Health Emergency in all national health system establishments,” including coordination of the national pandemic emergency led by the vice president of the republic with technical experts and the decentralized autonomous governments. On March 16, the national borders were closed. Subsequent measures were the prohibition of large public events, including processions and religious celebrations in agreement with ecclesiastical authorities, and the closure of gyms, cinemas, theaters, concerts, and other entertainment venues and events. On March 23, the government suspended class attendance for students at all levels. By early April, the health system was overwhelmed and many of Ecuador's COVID-19 deaths were reported in the province of Guayas, where corpses were abandoned on the street because local funeral homes were incapable of handling so much work. The government had to implement contingency measures for management of the deceased in Guayaquil, including cardboard coffins and the construction of emergency cemeteries. The government declared a “state of exception,” with support from the armed forces, to suspend Ecuadorians’ fundamental rights such as the freedoms of transit, assembly, and information. Limited testing capabilities for COVID-19 have hampered Ecuador’s response to the pandemic and limited tracking of the real magnitude of the pandemic in the country.

### Mexico.

The first case of COVID-19 was reported in Mexico on February 28 in the midst of a health sector reform in which a noncontributory social insurance scheme for low-income families (Seguro Popular) is transitioning into the new arrangement (Institute of Health for Welfare). On March 18, *The General Health Council*, chaired by the president and which includes federal and state administrative authorities, heads of health authorities in each Mexican State, and representatives from related public and private organizations, was established to guide the response. Until March 23, when all confirmed cases were imported or related to their contacts, interventions aimed at containing the epidemic focused on social distancing, avoidance of physical contact, individual hygiene measures (hand washing and use of alcohol gel), self-isolation if symptomatic, and special care for adults aged 60 years and older and for people with chronic illnesses. From March 23 to April 19, one of the most important health education efforts of the pandemic in Mexico, the “National Healthy Distance Campaign,” was launched. The campaign promoted maintenance of a distance of at least 1.5 meters between people through the character “Susana Distancia.”

On March 26, after community transmission was identified, additional mandatory restrictions were introduced. These restrictions were to suspend classes in all public and private schools, stay at home (except for essential outings for healthcare and food purchases), cancel public and private events, end meetings of more than 100 people, self-isolate if symptomatic, and quarantine for contacts for at least 15 days.

Although Mexico successfully managed the H1N1 “swine flu” pandemic in 2009, the response to COVID-19 has not been as rapid and impactful partly because of major changes in transitioning from Seguro Popular to a new health financing model; limited testing, which has probably understated the number of infections and deaths during the pandemic; corruption allegations related to the purchases of medical equipment; conflicting messages from health officials; and the president, who has minimized the importance of the pandemic and not followed the instructions (e.g., using face masks himself) of his technical collaborators and the General Health Council that he presides.

### Peru.

From 2017 to 2020, Peru experienced a period of political crisis and instability, which included the change of seven ministers of health. The president’s policies were resisted by the opposition party that held the majority in Congress. He was replaced by his vice president in March 2018, now President Vizcarra, who dissolved the Congress and called new elections, which took place in January 2020. Peru began its pandemic response preparations early, aware of the weaknesses of its fragmented and underbudgeted health system and the political instability.

On February 2, the Ministry of Health approved the National Plan for Preparation and Response against COVID-19 and a guideline for the management of suspected cases. However, there were delays and difficulties in purchasing PPE supplies, medical equipment, oxygen, and supplies for laboratory molecular testing.

On March 6, the first confirmed case of COVID-19 in Peru was reported. Five days later, the government delayed the start of the academic year and ordered travelers from China, France, Italy, and Spain to be quarantined at home or in hotel rooms for 14 days, but it was not strictly enforced. On March 13, universities were closed, public events with more than 300 people were banned, and social distancing and remote work were promoted. On March 15, the president declared a state of national emergency with a lockdown that placed strict controls on citizens’ movements, except to purchase food or pick up medicines. Remote work was introduced, and only workers from critical sectors were allowed to commute. National borders were closed the following day. Unfortunately, restrictions were not followed by all citizens, and on March 18^,^ the government began to enforce the measures and a nightly curfew from 8 pm to 5 am nationally, with support from the police and the military. By March 30, the number of people detained at police stations for breaking the curfew reached 33,000, much higher than the reported number of infected people at the time.^27^ Additional measures were implemented in the following weeks, such as to wear face masks when outside the home, to restrict movement to specific days by gender just to purchase groceries and medications. However, those measures were difficult to maintain. The Peruvian government also released several economic and social protection measures to address the emergency.^25^

The president has played an important leadership role throughout, informing the population each day at noon about the numbers of cases and the measures taken. However, there was never a nationwide strategic communication plan to guide communication actions or health education or a mass public awareness campaign to motivate people to protect their health or change their behavior and comply with preventive measures.

A shortage of molecular tests and laboratory capacity created initially difficulties in rapidly scaling up testing to diagnose new cases and contacts, prompting the government to purchase serologic tests, which were validated and swiftly deployed to scale-up testing.^28^

## WHAT WORKED WELL, WHAT HAMPERED THE RESPONSE, AND WHAT NEEDS TO BE ADDRESSED TO IMPROVE THE RESPONSE?

An important feature of the response in LA countries studied was the speed at which the countries were able to introduce voluntary and mandatory public health and disease control prevention policies, including population lockdowns, quarantines, and curfews, learning from international experiences. These countries were able to combine a public health response with economic and fiscal policies to soften the socioeconomic impact of the pandemic on low-income and self-employed groups.^25^ Supply side constraints for key components of the public health and health system response, including testing supplies, PPE for healthcare and related essential workers, and intensive care unit capacity, have hindered the response together with broader contextual factors (Table 2).

**Table 2:** Initial response to the COVID-19 pandemic in eight selected Latin American countries: what worked well and what has hampered the response

	What worked well?	What has hampered response?
Argentina	Rapid application of quarantine measures, which made it possible to strengthen the capacities of hospital services; training of human resources, mainly in intensive care management; widespread communication to the population from the Ministry of Health of the nation; effective coordination between federal and provincial levels; effective coordination between the public and private sectors in health	The national health system had suffered severe deterioration between 2016 and 2019, which required significant recovery efforts. This affected the economic situation of small and medium businesses, which implied strong orientation and negotiation efforts
Brazil	Strong epidemiologic and health surveillance system and online case notification system	Capacity constraints for large-scale molecular and antibody testing and ICU beds
	Rapid construction of field hospitals and conversion of existing hospital beds to create surge capacity	Political tensions when the Minister of Health was relieved of his position by the president because of public disagreement on the nature of restriction measures on commercial businesses
Chile	Chile has achieved relatively low death rates and established a “dynamic” quarantine. Hospital beds, including intensive care beds, have been available	The quarantine and lockdown measures have not been on a national scale, and local and regional policies have varied. The number of health workers infected has increased because of insufficient personal protective equipment
		The delay between testing, results, and medical leave for symptomatic cases poses risks for transmission. There is a need for more PCR testing in at-risk populations
Colombia	Rapid scale-up of laboratory and testing capacity and capacity of ICU beds	Political tensions due to the lack of coordination for quarantine orders between the national government and mayors and governors, which generated confusion among the population
		Opposition of the national government to curfews introduced by some mayors and governors
	A national fund established by civil society and different organizations to support low-income populations, the unemployed, and those working in the informal sector with precarious earnings	
		Social distancing, isolation, quarantine, and other voluntary restrictions are not consistently observed in some populations
Costa Rica	The Ministry of Health has played a political, strategic, and technical role through the response to formulate and articulate interventions with explicit agreement between the public and private sectors. Financial support for those experiencing job losses has mitigated the economic and social impacts of the epidemic	Restrictions create major challenges for people looking for jobs and generating income for their households
		Supply shortages for testing kits, respirators, personal protective equipment, and hospital capacity
		Increase in social pressure on the government to ease restriction measures from citizens experiencing “behavioral fatigue”
Ecuador	Introduction of a national emergency coordination of the pandemic led by the vice president of the republic with technical experts and the autonomous decentralized governments	Strong economic problems due to a recessive and dollarized economy, government management characterized by improvisation, and budget cuts for investment in health. A free trade agreement with the European Union from 2016 deepens the crisis
Mexico	The appointment of an undersecretary of health with strong technical–scientific skills for management of the pandemic, who is also directly involved in communications about the pandemic’s progression on a daily basis (press conferences)	The pandemic has coincided with major health reforms to replace Seguro Popular, which provided health insurance for low-income groups, leaving gaps in health services
		A large proportion of the population, who have informal employment with precarious income, have not been able to follow social distancing, isolation, and stay-at-home orders in the absence of income support and welfare measures
		Levels of crime and violence, generally against women and health personnel, have increased since the restrictions were introduced
		Political tensions due to contradictory messages related to the pandemic response from the General Health Council, federal health authorities, state governments, and the president
Peru	Leadership of the president to prioritize the health of the nation and introduce a rapid national response. Economic and financial support to lessen the socioeconomic impact on low-income groups. Introduction of rapid serologic tests allowed the scale-up of testing not only to symptomatics and contacts but also to other populations (military, police, health workers, and even people at markets). This led to a rapid increase in the number of “infected” people, including those positive on rapid serologic or molecular tests, which is hard to compare with other countries with more restrictive definitions of “confirmed cases of COVID-19,” which usually includes only those positive on molecular tests	Weak and fragmented health system with low capacity for molecular testing, isolation, and contact tracing, as well as ICUs and problems with oxygen availability
		High rates of informality and poor housing conditions with overcrowding
		Lack of a disease prevention and control communication plan
		Migration from urban areas like Lima, where the majority of Peru’s cases have been reported, to other regions and rural areas
		Mortality reported by Peru is not comparable with other countries because it includes deaths with any positive test to COVID-19, either molecular test or a serologic test

ICU = intensive care unit.

Governments face the major challenge of balancing the introduction of compulsory and voluntary restrictions on population movement and other efforts to slow and prevent disease transmission while trying to contain the adverse impact of the pandemic on their economies and populations, especially the financial and social fallout for the large proportions of their populations who are self-employed or work in the informal sector with few social protections. For a region that has the worst income inequalities in the world, the adverse social and economic impacts of the pandemic are likely to be large.

A major risk that needs to be addressed relates to the inconsistent compliance with social distancing, usage of face masks, isolation, quarantine, curfews, and other voluntary restrictions on movement that could lead to uncontrolled re-emergence of infections. A further risk relates to the potential spread of SARS-CoV-2 infections to indigenous populations, especially the isolated groups in the Amazon.

## HOW WILL COVID-19 IMPACT LATIN AMERICA?

The pandemic is far from being over, in the world and in our region. Latin America will be severely impacted by the global economic crisis that will follow the pandemic because of declines in economic activity with principal trading partners, especially China, falling commodity prices, interruption of global and regional value chains, a steep drop in the demand for tourism, and increased capital outflows from the region due to risk aversion in financial markets. We cannot ignore other impacts on public health such as possible increases in unintended pregnancies, mental health challenges, and domestic violence or worsening of chronic conditions due to overburdened health systems.

These changes will likely bring high unemployment, devaluation of currencies, and inflation. The economic crisis will be followed by social and public health consequences, with adverse effects due to foregone care for chronic diseases. This pandemic highlights the need to work on regional channels to coordinate the procurement of supplies; to strengthen regional intergovernmental cooperation on research, surveillance, and control; to effectively articulate the region’s public and academic health institutes and laboratories; and to provide effective training of human resources to be able to better address future public health challenges in the region.

## Supplemental material

Supplemental materials
