# Supplementary material for: COVID-19 Response in Latin America
Source: Am J Trop Med Hyg. 2020 Sep 15;103(5):1765–72. doi: 10.4269/ajtmh.20-0765 (PMC7646820; doi:10.4269/ajtmh.20-0765)
Supplement: Supplementary file 1 [file tpmd200765.SD1.pdf]

Supplemental Material 1: Initial mandatory restrictions for the COVID-19 Pandemic introduced by Argentina, Brazil, Chile, Colombia, Costa Rica, Mexico and Peru*

	Social distancing	Remote work	Closure of schools and universities	Ban on social and religious gatherings	Closure of shops, entertainment, leisure and meeting places	Closure of public transportation	Closure of economic activities	Lockdown	Curfew	Closure of international borders
Argentina	Stay at home and social distancing (9)	Yes (9)	Nationwide, public and private (12)	All gatherings (9)	All (9)	Partial. Social distancing promoted (17)	Yes, except for food and medicines (9)	Yes (9)	No	Yes (13)
Brazil	Stay at home and distancing of 1.5-2 metres (26)	Yes (20)	Nationwide, public and private (20)	Initially gatherings of >100, then >10 people (20)	Nationwide (27)	No. Reduced in some cities	Some commercial activities and offices. Not industrial or agricultural activities (27)	No	No	Land borders, except with Uruguay (25).<br>Air travel with selected countries (Europe and Asia) (28)
Chile	Stay at home and distancing of 1 metre (10)	Voluntary	Initially In regions with a high number of cases (12), now nationwide	Events with > 50 people (15)	Yes (15)	No	No	Yes (22)	Yes 10:00pm-5:00am (22)	Yes (13)
Colombia	Stay at home recommended. Social distancing – targeting persons over 70 years (4)	Yes (38)	Nationwide, public and private (10)	Initially events with >500 people (6), then >50 people (11)	All except hotels and shops selling food. Restaurants can deliver (12)	No	Yes (38)	No	No	Yes (11)
Costa Rica	Social distancing and avoidance of unnecessary movement (3)	Yes (3) but partial closure of certain economic activities	Nationwide, public and private (10)	50% reduction of the approved capacity in public meeting spaces (6), then banned (28)	Yes (6)	No	No. Partial closure of certain activities	No	No	Yes (12)
Mexico	Stay at home and distancing of 1.5 metres (24)	Encouraged, for non-essential activities (24)	Nationwide, public and private (18)	Initially gatherings of >100 people(24), then >50 people (31)	Nationwide (31)	No	Encouraged, for non-essential activities (24)	No	No	No
Peru	Stay at home and distancing of 1.5-2 metres (7)	Yes (7)	Nationwide, public and private (5)	Gatherings of >100 people (7)	Yes (10)	Yes (10)	Yes. Public services reduced, with introduction of remote services. (12)	Yes (10)	Yes 8:00pm to 5:00am, then 6:00pm to 5:00am (12).<br>Sundays all day.	Yes (10)

*(n) days after first case
